# The Efficacy of Extended Metacognitive Training on Neurocognitive Function in Schizophrenia: A Randomized Controlled Trial

**DOI:** 10.3390/brainsci12030413

**Published:** 2022-03-21

**Authors:** Can Wang, Yue Chong, Jiechun Zhang, Yili Cao, Yanbo Wang

**Affiliations:** 1 Shanghai Pudong New Area Mental Health Center, School of Medicine, Tongji University, 165 Sanlin Road, Shanghai 200124, China; 13917168835@163.com (C.W.); zhangjch@shspdjw.com (J.Z.); caoyl@shspdjw.com (Y.C.); 2 School of Medicine, Tongji University, 500 Zhennan Road, Shanghai 201802, China; cyevanism0824@163.com; 3 Division of Medical Humanities and Behavioral Sciences, School of Medicine, Tongji University, 500 Zhennan Road, Shanghai 200092, China

**Keywords:** metacognitive training, schizophrenia, metacognition, cognitive rehabilitation training

## Abstract

The aim of this study was to evaluate the effect of metacognitive training (MCT) on improving the neurocognitive function of Chinese patients with schizophrenia. One hundred inpatients with schizophrenia were selected by regional group randomization and divided into the control (treated as usual, TAU) group (*n* = 50) and the TAU + MCT group (*n* = 50). In this study, a 10-module MCT was used and the intervention process lasted 30 days. Cognitive function was assessed blindly using the Repeatable Battery of Neuropsychological Status (RBANS) scale at baseline, 24 h post-treatment, and 12 weeks’ post-treatment. The differences between the total RBANS score and baseline (pre-test) for the post-test and 12-week-follow-up tests were used as the primary outcome, and the difference between the RBANS dimension scores and baseline (pre-test) were used as a secondary outcome in this study. The completion rate at follow-up was high in the TAU + MCT group (94%). Intention-to-treat analysis and per-protocol analysis showed a significant increase in total neurocognitive function scores and three-dimensional scores (delayed memory, visual breadth, and attention) in the TAU + MCT group immediately after the intervention and at the 12-week follow-up compared with baseline. This study provides support for the efficacy of 10 module MCT concerning neurocognition.

## 1. Introduction

Schizophrenia is a complex and severe mental disorder [1] that is a major cause of disability worldwide [2]. People with a diagnosis of schizophrenia die an average of 10–25 years earlier than those without schizophrenia [3,4]. Clinically, schizophrenia is characterized by a heterogeneous range of symptoms classified under the following domains: positive symptoms, negative symptoms, cognitive impairments, and affective symptoms [5,6]. Cognitive impairments often appear before the onset of psychosis [7], and the decline in cognitive performance may be sustained through the chronic stages of schizophrenia, even in middle age [8]. Cognitive impairments are considered one of the most disabling symptoms and one of the best predictors of long-term functional disability [9].

Most patients with schizophrenia experience a broad range of cognitive impairments in neurocognition and social cognition [10]. The key domains of neurocognition that have been studied in schizophrenia include processing speed, attention/vigilance, working memory, verbal learning and memory, visual learning and memory, reasoning and problem solving, verbal comprehension, and verbal fluency [10]. Neurocognitive impairments are largely independent of positive psychotic symptoms and are seen as a core feature of the disorder [11]. Antipsychotics are the most widely used treatment for schizophrenia, but both first- and second-generation drugs provide insufficient neurocognitive enhancement and may even possess neurocognitive side effects [12,13]. Consequently, it is necessary to identify non-drug intervention measures for the neurocognitive function of patients with schizophrenia.

Cognitive rehabilitation holds an important place in non-pharmacological interventions for cognitive functioning in schizophrenia. Cognitive rehabilitation has been defined as “the therapeutic process of increasing or improving an individual’s capacity to process and use incoming information so as to allow increased functioning in everyday life [14]. This includes cognitive behavioral therapy and cognitive remediation, which use the plasticity of the cerebral cortex to promote behavioral improvement in neuropsychiatric disorders. In recent years, it has been increasingly used to improve cognitive function in schizophrenia [15,16,17]. Metacognitive training (MCT) [18], a novel and widely used group intervention for patients with schizophrenia, is particularly targeted at reducing cognitive bias [18]; it may be regarded as a hybrid of cognitive behavioral treatment and cognitive remediation. Metacognition is an extensive mental activity that involves the contemplation of one’s own thinking or others’ mental states [19]. Metacognitive deficits have been reported in all phases of schizophrenia and are related to poor treatment outcomes [20,21]. MCT aims to increase cognitive awareness (meta-level) by providing psychoeducation on cognitive biases followed by specific, structured cognitive exercises. Several studies have confirmed that MCT is an effective form of therapy for reducing the positive symptoms of schizophrenia (especially delusions), cognitive biases, and for improving cognitive insight and general functioning [22,23,24,25,26]. The original MCT contained 8 modules, and the extended MCT added 2 modules to this, for a total of 10 modules.

MCT involves many cognitive tasks similar to cognitive remediation [27,28], which already shows some neurocognitive effects [29]. Single studies investigating the effect of MCT on neurocognition in schizophrenia are scarce and yield promising but also divergent results. Inconsistent effects have been reported for neurocognitive subdomains including memory [30,31] and selective attention [30,32], possibly because of methodological differences, such as outcome measures and cognitive subdomains assessed, intervention type, and choice of control or comparison intervention [33]. The treatment process of MCT for cognitive bias potentially utilizes memory, working memory, and executive monitoring processes, and some improvements in neurocognitive function may reflect a link between cognitive bias and neurocognition [34]. Shan X et al. [35] showed that after eight weeks of MCT treatment, memory and attention were significantly improved in the intervention group, and scans performed by resting-state functional magnetic resonance imaging revealed an increase in regional homogeneity in the participants’ right precuneus. The precuneus is involved in episodic memory retrieval, visuospatial imagery and spatial navigation [36], and plays an especially important role in memory metacognition, such as episodic memory retrieval [37].

The aim of this study was to investigate the neurocognitive effects of extended MCT in schizophrenia. We hypothesized that participants who received MCT would show improvements in neurocognition.

## 2. Materials and Methods

### 2.1. Procedure and Design

**Figure 1:**
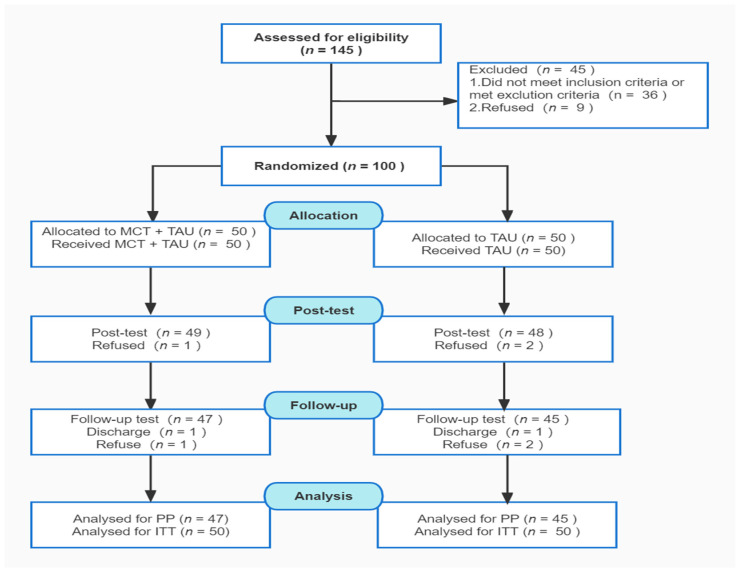
CONSORT flow chart.

This study was a prospective, 1-center, single-blinded, randomized clinical trial with a parallel 2-arm group conducted in China. After a baseline assessment, patients were randomly assigned to either the “TAU” (treated as usual) condition or the “TAU + MCT” condition in a 1:1 ratio (see Figure 1). Random assignment was performed by independent investigators who did not have access to patient information. Follow-up assessments were performed at 24 h and 12 weeks after the end of the MCT program. The evaluators were blind to treatment assignment to prevent the Rosenthal effect. The study was approved by the Ethics Committee of Shanghai Pudong New Area Mental Health Center (PDJWLL20200016) and registered in the Clinical Trials Registry Platform (No. ChiCTR2100052421). This study followed the Consolidated Standards of Reporting Trials (CONSORT) reporting guidelines.

### 2.2. Participants

The participants were drawn from the inpatient department of the Shanghai Pudong New Area Mental Health Center from November to December 2020 according to the district group randomization method. The patient inclusion criteria were as follows: age between 18 and 55 years; a diagnosis of schizophrenia (ICD 10 criteria, F20–29) by a psychiatrist; psychiatric symptoms were basically stable, and the total score for the Positive and Negative Syndrome Scale (PANSS) was less than or equal to 60 points; participants level of education was junior high school or above; and patients were on a relatively stable medication regimen with second-generation antipsychotics. Those who had recently participated in cognitive behavioral therapy, had serious adverse reactions to antipsychotic medications, were diagnosed as having mental retardation, or had a combination of serious organic diseases were excluded. A total of 145 patients were initially recruited (see Figure 1). However, 45 patients were excluded for not meeting the inclusion criteria (screening-to-inclusion ratio = 69%). It should be pointed out that for schizophrenia patients in the inpatient department, the PANSS scale is a routine medical order to be assessed once a month, and is carried out by the attending doctor.

All participants were informed of the objectives, methods, benefits, and possible risks of the study. All patients signed a written informed consent form. Participant confidentiality was assured during all study procedures. Participants were informed that they could withdraw their participation at any time without penalty. Only members of the study team and the health professionals responsible for the care of the participants had access to the participants’ data.

### 2.3. Sample Size

The difference between groups with a medium effect size (f = 0.25) was specified as clinically significant. With α = 0.05, power = 0.80, and a correlation between repeated measures of 0.5, the sample size obtained using the G*Power3.1.9.2 software was 86 cases, and considering a 10% dropout rate, at least 96 cases needed to be included at baseline.

### 2.4. Data Collection and Outcome Measures

Psychometric instruments were applied by interview to all participants in the study at three different assessment times (before the first treatment (baseline), after the last treatment (post-treatment), and 12 weeks after the last treatment (follow-up)). Neurocognitive function was assessed using psychometric tools in all subjects. In addition, sociodemographic data (age, sex, duration of mental disorder, number of psychiatric hospitalizations) were collected at baseline.

#### 2.4.1. Primary Outcomes

This study used the difference between post-intervention (post-test and 12-week-follow-up test) and baseline cognitive function scores as the primary outcome. Cognitive function was assessed using the Repeatable Battery of Neuropsychological Status (RBANS).

#### 2.4.2. Secondary Outcomes

This study used the difference between post-intervention (post-test and 12-week-follow-up test) and baseline RBANS dimension (immediate memory, visual–spatial structure, verbal function, attention, delayed memory) scores as the secondary outcomes.

#### 2.4.3. Outcome Measures

This study used the Repeatable Battery of Neuropsychological Status (RBANS) [38], a structured cognitive function assessment scale that is simple, rapid, and valid. The entire battery takes less than 30 min to administer, with minimal materials required and the extent and pattern of cognitive impairment are broadly consistent with the findings of more comprehensive neuropsychological batteries [39,40,41]. The Chinese version of the RBANS has good reliability and validity in schizophrenic patients and can be used as a screening tool for cognitive functioning in schizophrenic patients [42]. The RBANS test consists of 12 subtests, including 5 dimensions: (1) immediate memory, including vocabulary recall and story retelling, which examines subjects’ ability to memorize and recall vocabulary and long sentences in a short period of time, with 1 point for each correct retelling, for a total of 64 points; (2) visual-spatial structure, including two tests, graphic copying and line orientation, which examine subjects’ ability to perceive and manipulate visually within a limited period of time, with 1 point for each correct copy or answer, for a total of 20 points; (3) verbal function, including two tests of picture naming and semantic fluency, which examines subjects’ abilities in verbal comprehension and fluent expression within a time limit, where one correct answer is one point, for a total of 50 points; (4) attention, including two tests of numerical breadth and coding, which examine subjects’ auditory and visual attention within a time limit, with two points for correct numerical breadth on the first test, one point for correct numerical breadth on the second test, and zero points if both tests were incorrect; (5) delayed memory, with four tests including vocabulary recall, vocabulary reacquaintance, story recall, and graphic recall, which are administered to examine the correct recall rate for the first and second parts of vocabulary and graphic recall, with one point for each correct recall for a total of 62 points. The final total score was processed by standard score conversion.

The RBANS test is divided into 2 equivalent versions. In this study, the A and B versions were assessed at intervals to reduce learning effects, with the A set of tests used for the baseline assessment and the 12-week post-intervention assessment, and the B set of tests was used for the assessment immediately after the end of the intervention. Because the two versions were not considered fully equivalent in terms of language factors and the difficulty of the B version was higher than that of the A version, the equivalence was adjusted by adding 4 points to the original verbal fluency score of the B version and then converting the standard score as described in the original operational manual.

All assessors received formal training on the test, with good inter-assessor measurement agreement (kappa > 0.8, intragroup correlation coefficient > 0.75).

At the end of the MCT sessions, satisfaction with treatment was assessed using the MCT Satisfaction Scale. This scale was previously used in a study on the MCT [43]^,^ and the internal consistency was found to be satisfactory (Cronbach’s alpha = 0.73). The measure contained 10 items covering distinct aspects of training satisfaction: effectiveness, usefulness, applicability to daily life, transparency of the aims, and fun. Each item was rated on a 5-point scale (1 = do not agree at all, to 5 = totally agree). Higher scores indicated greater satisfaction.

### 2.5. Study Intervention

#### 2.5.1. Control Group

TAU consisted of conventional psychiatric medication and a supportive rehabilitation program that included psychoeducation and social activities. The control group did not participate in the MCT programs in this trial.

#### 2.5.2. Intervention Group

In this condition, patients underwent MCT in addition to TAU using a Chinese version of MCT (see Metacognitive Training for Psychosis—Clinical Neuropsychology Unit (https://clinical-neuropsychology.de (accessed on 21 March 2022)) with eight standard and two additional modules.

The topics for MCT included the following: attribution blaming and taking credit (Module 1), jumping to conclusions (Modules 2 & 7), changing beliefs (Module 3), deficits in theory of mind and social cognition (Modules 4 & 6), overconfidence in (memory) errors (Module 5) and depression and low self-esteem (Module 8), improving low self-esteem (Module 9), and normalization and ways to deal with stigma (Module 10). Each module consisted of one session. Each module was supported by multimedia slides and homework exercises. Additional support materials such as videos could also be used. All these materials are available at www.uke.de/mct (accessed on 21 March 2022).

All MCT was delivered as a group intervention. The number of patients in each group in this study was 10. According to the MCT manual guidelines (see www.uke.de/mct (accessed on 21 March 2022)), it takes 30 days to complete the 10 modules by completing 1 metacognitive training module every 3 days, lasting 45–60 min each time. MCT is an open program, so patients can join at any time during each cycle. During each cycle, if a participant misses a session, it does not need to be repeated. The MCT is not designed to require modules to be completed sequentially.

The intervention team included the person performing the MCT, quality control staff, and supervisory staff. The personnel implementing the intervention received appropriate prior training. The quality control officer was a chief psychiatrist with extensive clinical experience who was responsible for assessing the quality of the MCT implementation and providing feedback to the implementer. The supervisor was a doctor of psychology, and the intervention team members regularly discussed, checked, and improved the quality of implementation under the guidance of the supervisor. Furthermore, the same investigator administered the questionnaires at all three time points and had clinical and research experience in treating and investigating schizophrenia. All data were collected through face-to-face interviews.

### 2.6. Statistical Analyses

At baseline, group comparisons were conducted using the independent-sample *t* test for normally distributed continuous variables, the Mann–Whitney U test, where the assumption of normal distribution was violated, and the chi-square test for categorical variables. Continuous variables are expressed as the means with SDs or medians with interquartile ranges, and mean differences are expressed with their 2-sided 95% CIs. The analyses were conducted by assuming both a per-protocol (PP) and an intention-to-treat (ITT) strategy. The PP analyses considered participants who completed the three assessments (baseline, post-treatment and follow-up). The ITT analyses considered data from all participants whose baseline data were available. Multiple imputation was adopted to estimate post-treatment and follow-up scores for non-completers.

The primary outcome was calculated using analysis of multivariate covariance controlling for baseline scores (MANCOVA). Differences between cognitive function on the follow-up tests were assessed at 24 h and 12 weeks after the end of the intervention and baseline as dependent variables, with the treatment condition as a fixed factor and the baseline score as a covariate. This strategy has been used in previous MCT efficacy studies [32]. The partial η^2^ statistic was calculated as an effect size. The level of significance was set as a 2-sided *p* value less than 0.05. All analyses were conducted with SPSS version 26.0 (SPSS Inc.).

## 3. Results

### 3.1. Baseline Characteristics

At baseline, no differences were observed between groups in terms of sex, age, number of hospitalizations, duration of disease, total RBANS score, or each dimensional score (Table 1).

### 3.2. Primary Outcomes

The outcomes by treatment condition (TAU + MCT vs. TAU) and MANCOVA results are presented in Table 2. Taking the ITT analysis as an example, the MANCOVA revealed a significant group difference in support of MCT, which emerged during baseline-post (F = 44.846, ηp^2^ = 0.316, *p* < 0.001) and baseline-follow-up tests (F = 83.356, ηp^2^ = 0.462, *p* < 0.001). Therefore, the improvement in cognitive function (total RBANS score) was significantly greater in the TAU + MCT group than in the TAU group. The PP and ITT analyses did not differ (i.e., for all analyses, the level of significance (*p* < 0.05) remained unchanged).

### 3.3. Secondary Outcomes

For visual–spatial, attention, and delayed memory scores, the TAU + MCT group was superior to the TAU group at both post-treatment and follow-up time points. For the immediate memory score and language score, no results were significant. Again, there was no difference between the PP and ITT analyses (Table 3).

### 3.4. Completion and Subjective Assessment of the Training

Completion at the 12-week-follow-up was high (TAU + MCT, *n* = 47, 94%; TAU, *n* = 45, 90%). Most parameters were positively appraised by the participants (see Table 4). Most participants rated the MCT as useful and sensible and would recommend the training to others.

## 4. Discussion

The current trial is the first implementation of a 10-module MCT program in a Chinese population to assess its impact on neurocognitive functioning in hospitalized patients with schizophrenia. Our study provides support for the efficacy of 10 modules of MCT concerning neurocognition.

In our study, the TAU + MCT group showed significant improvement in delayed memory, consistent with the results of previous studies [30,31]. However, no improvement in immediate memory was found in our study, which is inconsistent with the findings of Moritz et al. [30] and consistent with the results of two other studies [31,32]. One of the MCT modules conveyed strategies for improved learning (for instance, mnemonic aids, involving multiple sensory channels for recollection) that may have an impact on improving memory. Verbal memory is associated with lower self-reflection and understanding of others’ psychology in schizophrenia (SZ) patients. This was the training theme of MCT module 3, module 4, and module 6, which may also explain the improvement in delayed memory. Although it is speculative at this point, the attenuation of distress may have released cognitive resources that were previously occupied by ruminative and paranoid thoughts. In addition, information processing speed was found to predict SZ distant memory impairment [44]. In the present study, improvement through information processing speed may have led to a subsequent improvement in memory for words and graphics in the TAU + MCT group.

The attention dimension in RBANS consists of two tests, numerical breadth and coding. These tests examine subjects’ auditory attention and visual attention within a time limit and are often used to measure individuals’ speed and attention in cognitive processing, which may be one of the most severe neuropsychological deficits in schizophrenia [45]. In our study, the TAU + MCT group showed a significant improvement in attention compared to the TAU group, which is different from the results of several studies [32,46,47]. The findings of Moritz S et al. [31] are consistent with our study, in that they assessed participants’ choice of attention and found significant differences between groups (*p* = 0.04, η^2^ = 0.053). It has been suggested that the use of relevant memory strategies in SZ can improve patients’ deficits in cognitive processing speed and attention. In this study, we used a strategy to improve memory learning to train patients’ memory, encouraged them to apply it to their daily lives, and provided feedback to the trainers on the problems that occurred during application to enable targeted instruction. The improvement in patients’ attention may be related to this, in line with the findings of Bachman et al. [48].

The visual-breadth dimension in RBANS was used to examine subjects’ visual perception and manipulation abilities for a limited period of time. The TAU + MCT group showed significant improvements compared to baseline, both post-treatment and at the 12-week-follow-up. Deficits in executive functioning are associated with lower levels of self-reflection and understanding of others’ psychology in schizophrenic patients, and improvements in the latter may affect subjects’ ability to perform operations. On the other hand, some potential psychological effects of MCT may surface once cognitive biases are stabilized. Effects on neurocognitive subdomains are inconsistent, possibly because of methodological differences such as the outcome measures and cognitive subdomains assessed, intervention types, and the choice of a control or comparison intervention.

## 5. Limitations

Some limitations of this trial should be acknowledged. First, social expectations are a potential limitation. Although the assessor in charge of the psychometric evaluation was unaware of the subgroups of the subjects, because they were employees within this health care institution, the participants attempted to please them by responding positively. To mitigate this limitation, the researcher conducting the psychometric assessment should ideally be an individual external to the institution. Second, the TAU group implemented a supportive rehabilitation program, including psychological education and social activities. We did not control for these effects. Third, we restricted participants to those under age 55 to control for the potential of organic brain disorders, which may limit our findings because our age range was different from previous studies. Finally, subjects in this study were required to have a junior high school education or above, but recent meta-analyses suggest that people with lower levels of education actually benefit more from cognitive remediation, and perhaps further exploration is needed in the future.

## 6. Conclusions

This RCT tested the efficacy of a new 10-module MCT. Our findings suggest that extended MCT can improve neurocognitive function in patients with schizophrenia.

## Figures and Tables

**Table 1:** Baseline characteristics.

Characteristic	TAU + MCT *n* (%)	TAU *n* (%)	Statistics	*p* Value
Demographic data				
Sex			χ^2^ = 0.162	0.687
Male	23 (46.0%)	21 (42.0%)		
Female	27 (54.0%)	29 (58.0%)		
Treatment-related data				
Hospitalizations (including present)			χ^2^ = 1.296	0.523
Once	15 (30.0%)	15 (30.0%)		
1–5 times	25 (50.0%)	29 (58.0%)		
>5 times	10 (20.0%)	6 (12.0%)		
Years of disease			χ^2^ = 1.213	0.545
<5	17 (34.7%)	20 (40.0%)		
5–10	21 (42.9%)	23 (46.0%)		
>10	11 (22.4%)	7 (14.0%)		
	Mean (*SD*)	Mean (*SD*)		
Age (years)	44.66 (9.612)	44.34 (8.530)	t = 0.726	0.469
RBANS	70.00 (11.884)	69.84 (13.312)	t = 0.063	0.950
Immediate memory	73.64 (18.098)	70.96 (11.480)	t = 0.884	0.379
Visual spatial	77.12 (10.634)	77.32 (16.918)	t = 0.071	0.994
Language	78.22 (10.620)	78.28 (12.636)	t = 0.026	0.980
Attention	77.78 (13.146)	78.06 (12.369)	t = 0.110	0.913
Delayed memory	74.70 (18.010)	75.42 (17.182)	t = 0.205	0.838

**Table 2:** Group differences across time on measures of cognitive function.

Variables	Group	Mean (SD)			Per-Protocol Statistics Mean Differences (95% Cl) across Time and MANCOVAs		Intention-to-Treat Statistics Mean Differences (95% Cl) across Time and MANCOVAs	
		Baseline	Post-Treatment	Follow-Up	Baseline-Post	Baseline-Follow-Up	Baseline-Post	Baseline-Follow-Up
RBANS	TAU + MCT	69.81 (12.24)	74.51 (12.45)	76.36 (12.97)	4.702 (4.045–5.359)	6.556 (5.975–7.137)	4.755 (4.114–5.397)	6.490 (5.946–67.033)
	TAU	70.22 (13.97)	71.78 (14.16)	73.13 (13.94)	1.555 (0.884–2.227)	2.908 (2.314–3.502)	1.583 (0.935–2.231)	2.896 (2.347–3.446)
					F = 44.327, ηp^2^ = 0.332 *p* < 0.001	F = 76.028, ηp^2^ = 0.461 *p* < 0.001	F = 44.846, ηp^2^ = 0.316 *p* < 0.001	F = 83.356, ηp^2^ = 0.462 *p* < 0.001

**Table 3:** Group differences across time on measures of cognitive function dimensions.

Variables	Group	Mean (SD)			Per-Protocol Statistics MD (95% CI) across Time and MANCOVAs		Intention-to-Treat Statistics MD (95% Cl) across Time and MANCOVAs	
		Baseline	Post-Treatment	Follow-Up	Post-Treatment	Follow-Up	Post-Treatment	Follow-Up
Immediate memory	MCT	73.64 (18.10)	74.79 (17.19)	77.04 (17.79)	1.845 (1.016–2.647)	4.083 (3.096–5.070)	1.747 (0.946–2.549)	3.701 (2.993–4.408)
	TAU	70.96 (11.48)	72.58 (11.10)	74.73 (11.17)	1.384 (0.537–2.231)	3.558 (2.549–4.566)	1.279 (0.469–2.089)	3.014 (2.299–3.729)
					F = 0.595, ηp^2^ = 0.007 *p* < 0.443	F = 0.547, ηp^2^ = 0.006 *p* < 0.462	F = 0.665, ηp^2^ = 0.007 *p* < 0.417	F = 1.835, ηp^2^ = 0.019 *p* < 0.179
Visual–spatial structure	MCT	76.83 (10.75)	80.68 (11.44)	81.60 (11.76)	3.851 (3.164–4.538)	4.766 (4.030–5.502)	3.779 (3.112–4.447)	4.821 (4.152–5.490)
	TAU	77.60 (17.28)	78.96 (17.47)	79.60 (17.35)	1.356 (0.653–2.058)	2.000 (1.248–2.752)	1.308 (0.634–1.983)	1.975 (1.299–2.651)
					F = 25.520, ηp^2^ = 0.223 *p* < 0.001	F = 27.384, ηp^2^ = 0.235 *p* < 0.001	F = 26.715, ηp^2^ = 0.221 *p* < 0.001	F = 35.283, ηp^2^ = 0.273 *p* < 0.001
Verbal function	MCT	78.55 (10.57)	81.00 (10.91)	82.15 (10.37)	3.856 (3.167–4.545)	4.772 (4.034–5.509)	2.976 (2.010–3.941)	4.090 (3.145–5.035)
	TAU	79.42 (12.15)	81.22 (12.50)	82.38 (11.94)	1.350 (0.646–2.055)	1.994 (1.240–2.748)	1.775 (0.800–2.750)	2.866 (1.911–3.821)
					F = 1.277, ηp^2^ = 0.014 *p* < 0.261	F = 1.657, ηp^2^ = 0.018 *p* < 0.201	F = 3.017, ηp^2^ = 0.031 *p* < 0.086	F = 3.274, ηp^2^ = 0.034 *p* < 0.074
Attention	MCT	77.32 (13.38)	83.28 (13.97)	85.17 (14.28)	5.953 (4.913–6.993)	7.848 (6.825–8.871)	5.918 (4.919–6.917)	7.714 (6.741–8.687)
	TAU	77.60 (12.51)	79.67 (11.99)	80.60 (11.81)	2.071 (1.008–3.135)	3.003 (1.958–4.049)	2.084 (1.074–3.094)	3.021 (2.038–4.004)
					F = 26.879, ηp^2^ = 0.232 *p* < 0.001	F = 43.279, ηp^2^ = 0.327 *p* < 0.001	F = 28.715, ηp^2^ = 0.234 *p* < 0.001	F = 45.357, ηp^2^ = 0.325 *p* < 0.001
Delayed memory	MCT	74.98 (18.37)	80.96 (17.21)	82.55 (18.04)	5.956 (4.905–7.007)	7.556 (6.519–8.593)	5.975 (4.959–6.991)	7.527 (6.680–8.373)
	TAU	75.60 (17.01)	77.31 (16.28)	78.07 (15.88)	1.735 (0.661–2.809)	2.486 (1.426–3.546)	1.838 (0.812–2.864)	2.962 (2.107–3.817)
					F=31.132, ηp^2^ = 0.259 *p* < 0.001	F = 46.142, ηp^2^ = 0.34 *p* < 0.001	F = 32.364, ηp^2^ = 0.256 *p* < 0.001	F = 56.741, ηp^2^ = 0.376 *p* < 0.001

Note: MCT refers to the TAU + MCT group.

**Table 4:** Subjective assessment of the MCT interventions at post-treatment (*n* = 47).

	Yes	No
The training was useful and sensible.	47 (100%)	0
I had to force myself to go to the training regularly.	18 (17%)	29 (63%)
In everyday life, I do not apply the lessons learned.	20 (43%)	27 (57%)
The training was an important part of my treatment program.	43 (91%)	4 (9%)
I would have liked to spend the time doing something else.	15 (32%)	32 (68%)
The training was fun.	44 (94%)	3 (6%)
A lot of what I learned during training is useful for daily life.	44 (94%)	3 (6%)
The goals and rationale of the training were clear to me.	42 (90%)	5 (10%)
I would recommend the training to others.	47 (100%)	0
I found it beneficial that the training was administered in a group.	47 (100%)	0
